# A Case of Japanese Spotted Fever Coinciding With COVID-19 That Progressed to Septic Shock and Cardiac Arrest: A Case Report

**DOI:** 10.7759/cureus.80805

**Published:** 2025-03-19

**Authors:** Takuya Fusada, Nobuya Kitamura

**Affiliations:** 1 Department of Emergency and Critical Care Medicine, Kimitsu Chuo Hospital, Chiba, JPN

**Keywords:** acute kidney injury, coinfection, covid-19, intensive care, japanese spotted fever, rash, septic shock

## Abstract

Japanese spotted fever is a tick-borne infectious disease caused by Rickettsia japonica. Early treatment is crucial to prevent deterioration and death. We present a case of an 82-year-old male with Japanese spotted fever coinciding with coronavirus disease 2019 (COVID-19). Initially diagnosed with COVID-19 via a severe acute respiratory syndrome coronavirus 2 (SARS-CoV-2) antigen test at a clinic and advised to recuperate at home, the patient already exhibited a rash at the time of diagnosis. He later developed difficulty with oral intake and became lethargic. Upon visiting the emergency room, he presented with erythema with purple tints on the trunk and extremities, including palms and soles. Laboratory tests revealed acute kidney injury and hyperkalemia. Suspecting prerenal acute kidney injury due to inadequate oral intake during COVID-19 treatment, we initiated rehydration therapy. The rash was initially attributed to COVID-19, but dermatological examination suggested Japanese spotted fever based on its distribution on the palms and soles, which is atypical for COVID-19. Tick bites were observed on the right thigh, prompting initiation of minocycline. A subsequent polymerase chain reaction (PCR) test later confirmed Japanese spotted fever. The patient deteriorated, experiencing cardiac arrest. He required intensive care but recovered and was transferred to a convalescent rehabilitation hospital. This case highlights the potential for overlooking Japanese spotted fever due to an initial diagnosis of COVID-19. Recognition of distinct rash characteristics led to the correct diagnosis. A thorough physical examination remains crucial, even when a COVID-19 diagnosis has already been made.

This article was previously presented as a meeting abstract at the 51st Annual Meeting of the Japanese Society of Intensive Care Medicine on March 14, 2024.

## Introduction

Japanese spotted fever is a tick-borne infectious disease caused by Rickettsia japonica. First reported in 1984, its incidence has been increasing in recent years. The majority of cases occur on the Pacific side of Japan in and west of Chiba prefecture, but reports of cases in other regions have been increasing as the disease has expanded. Infection often occurs by outdoor activities that involve the chance of contact with ticks, and the incubation period is 2-8 days. The symptoms of the disease start with nonspecific ones such as fever and fatigue. Fever, skin rash, and a dark scab (eschar) at the tick bite site are considered as the triad of symptoms, although a study of a single region reported that only about 70% of cases had fever. The most common rickettsioses in Japan are Japanese spotted fever and Scrub typhus. Compared to scrub typhus, Japanese spotted fever has smaller eschar and a smaller rash, which is more prominent on the extremities, including the palms and soles. The reported mortality rate is approximately 1.1%, with a better prognosis than other rickettsial diseases. Early administration of tetracyclines is crucial, and international guidelines recommend initiating antimicrobial treatment immediately, without waiting for laboratory confirmation via polymerase chain reaction (PCR) testing [1-4].

Coronavirus disease 2019 (COVID-19), caused by severe acute respiratory syndrome coronavirus 2 (SARS-CoV-2), is highly infectious and has had a global impact. After the first reported case in Wuhan, Hubei Province, China, in late December 2019, the virus rapidly spread worldwide, leading the World Health Organization to declare a pandemic on March 11, 2020. COVID-19 has resulted in over 6 million deaths globally. Despite advances in prevention and treatment, the disease remains prevalent in many countries due to emerging variants [5]. The pandemic has altered healthcare practices, with providers prioritizing COVID-19 screening for febrile patients and often limiting detailed physical examinations to reduce patient contact time [6]. Additionally, cases of co-infection with COVID-19 and other infectious diseases have been reported. Co-infection may increase the risk of deterioration [7], and some cases have been misdiagnosed due to the prioritization of COVID-19 testing, which delays the diagnosis of other diseases [8-10]. Although rash is not a primary symptom of COVID-19, it occurs in approximately 20% of patients with various types of rashes; however, COVID-19-related rashes do not affect the palms and soles [11].

This case report describes an 82-year-old male with COVID-19 whose condition worsened during home treatment. A dermatological consultation led to the diagnosis of Japanese spotted fever, which progressed during hospitalization.

## Case presentation

An 82-year-old male was transported to the emergency room by ambulance due to impaired consciousness. His medical history included stroke, hypertension, type 2 diabetes mellitus, and chronic kidney disease. He regularly visits the mountains and had been in the mountains a week before admission. The area he lived was surrounded by mountains, and rickettsiosis occurred with some frequency. Five days before admission, he experienced fever, fatigue, and loss of appetite and visited a clinic. He was diagnosed with COVID-19 based on a positive rapid antigen test for SARS-CoV-2. Ensitrelvir was prescribed, and he was advised to recuperate at home. At the time of the medical examination, his son noticed a rash on his body.

**Figure 1:**
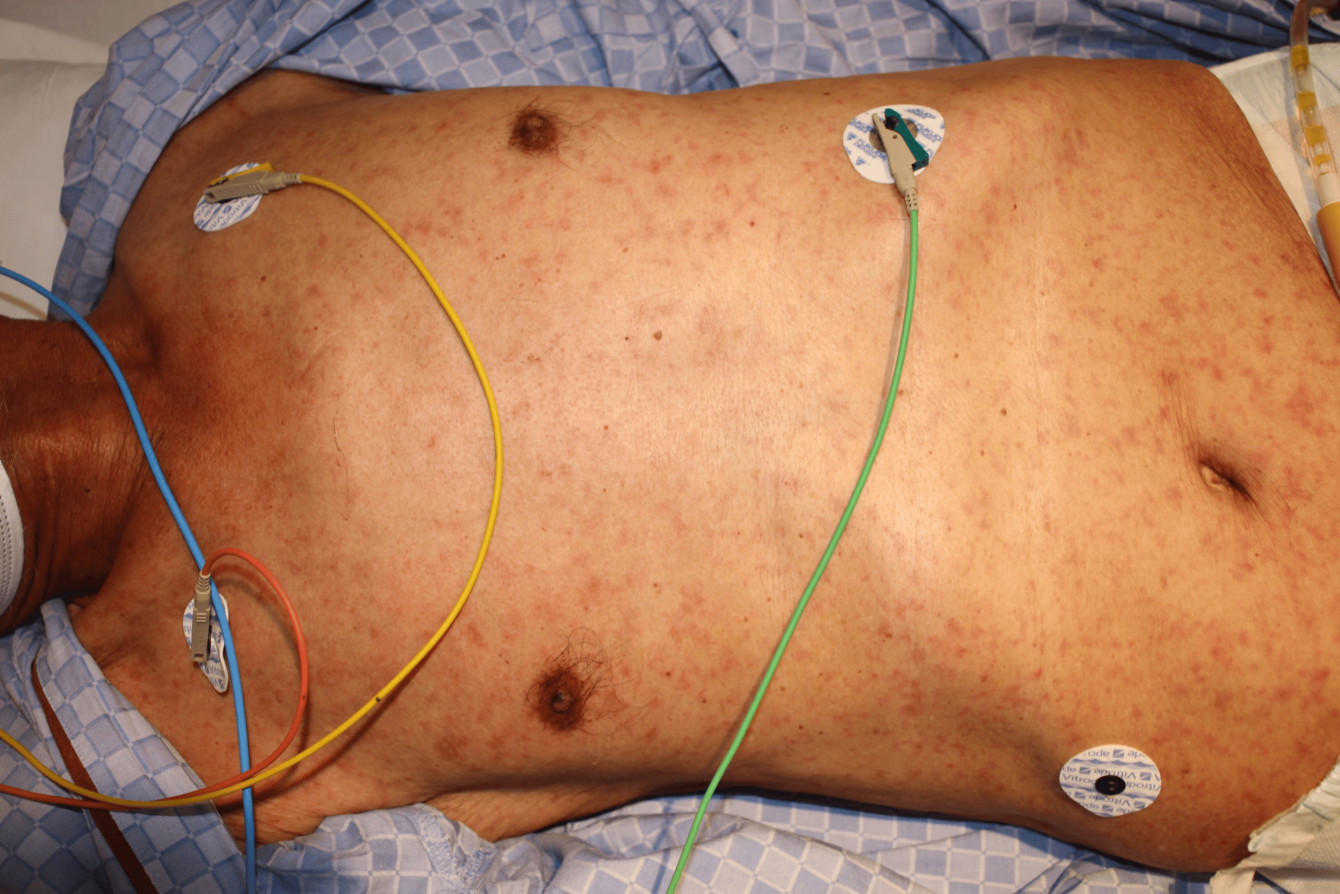
Rashes on the trunk on admission day Small rashes were widely distributed on the trunk. The rashes were considered as a kind of erythema because they disappeared on finger pressure, but the tints of the rashes were purple.

**Figure 2:**
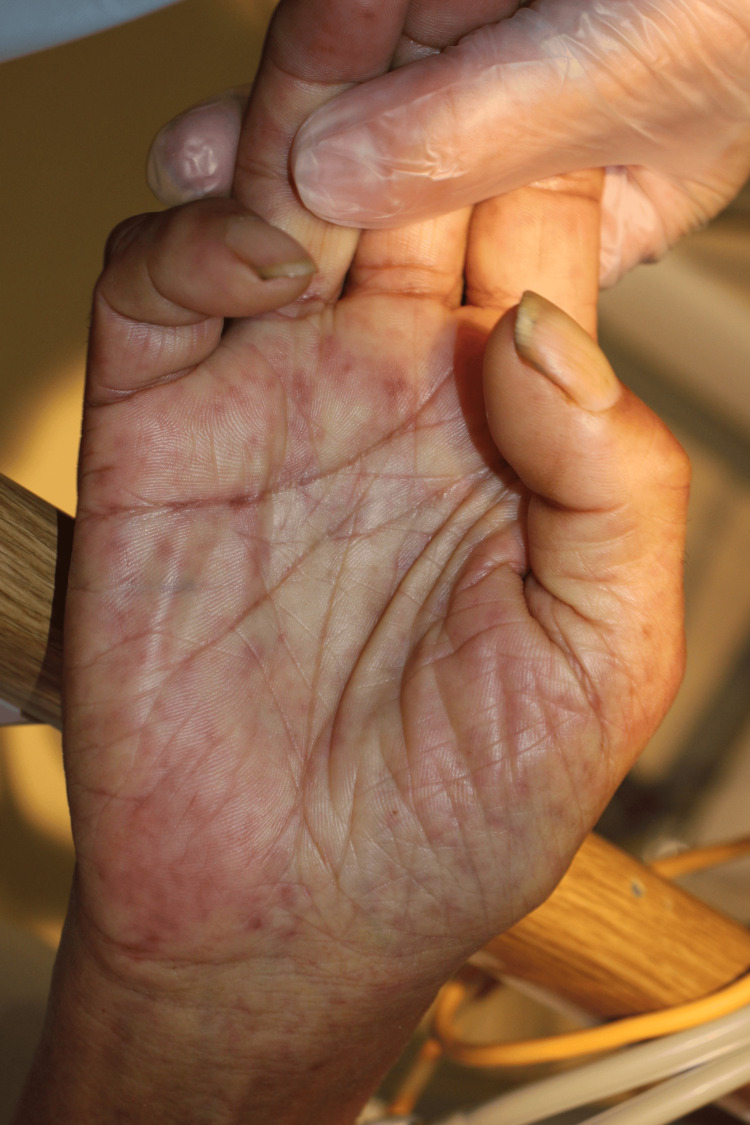
Rash on the palm on admission day A similar rash was observed on the palms, which was atypical for COVID-19.

**Figure 3:**
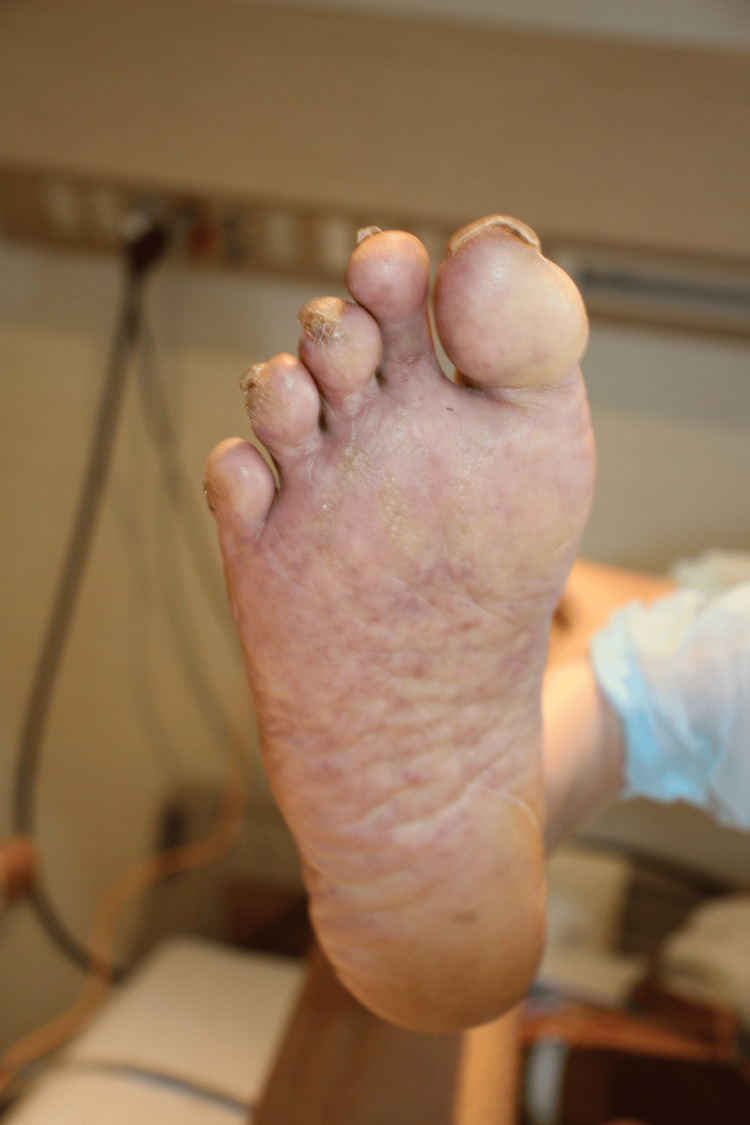
Rash on the sole on admission day Similar rash was observed on the soles, which was also atypical for COVID-19.

The patient was unable to eat any meals and, on the day before admission, had difficulty drinking water. On the day of admission, he became lethargic, prompting his son to call emergency medical services. His vital signs were as follows: temperature, 37.3°C; heart rate, 106 beats per minute; blood pressure, 98/61 mmHg; respiratory rate, 30 breaths per minute; and oxygen saturation, 97% on 10 L/min of oxygen via a non-rebreather mask. His Glasgow Coma Scale score was E3V4M6. Physical examination revealed rashes on the trunk and extremities, including the palms and soles (Figures 1-3). The rashes were considered as a kind of erythema because they disappeared on finger pressure, but the tints of the rashes were purple. He had no signs of pneumonia, respiratory symptoms, or hypoxia, even after supplementary oxygen was discontinued.

The SARS-CoV-2 PCR test for confirmation was positive. Laboratory analysis showed high creatinine level, hyperkalemia, severe metabolic acidosis and high levels of inflammatory markers (Table 1). Prerenal acute kidney injury due to oral feeding intolerance during COVID-19 treatment was suspected, and impaired consciousness was attributed to high urea nitrogen levels. The patient was admitted for rehydration. Remdesivir was not administered because it was not recommended for patients with severe renal failure in Japan at that time, and other antiviral drugs for COVID-19 were not available in our hospital. Similarly, other anti-COVID-19 therapies, such as steroids, were not used because the patient showed neither signs of pneumonia nor respiratory distress.

**Table 1:** Laboratory investigations on admission day

Test	Observed Value	Reference Range
White blood cell count	20,400 /uL	3,500–9,000 /uL
Hemoglobin	13.2 g/dL	13.5–17.6 g/dL
Hematocrit	41.30%	40–48%
Platelet count	146,000 /uL	150,000–350,000 /uL
Sodium	136 mEq/L	137–147 mEq/L
Potassium	7.4 mEq/L	3.5–5.0 mEq/L
Chloride	103 mEq/L	98–110 mEq/L
Urea nitrogen	190.3 mg/dL	8–21 mg/dL
Creatinine	8.67 mg/dL	0.65–1.07 mg/dL
C-reactive protein	18.2 mg/dL	0–0.3 mg/dL
Arterial pH	7.257	7.35–7.45
Bicarbonate	8.7 mEq/L	22.2–28.3 mEq/L
PaCO2	20.1 Torr	35–45 Torr
Lactate	4.3 mmol/L	0.5–1.5 mmol/L

**Figure 4:**
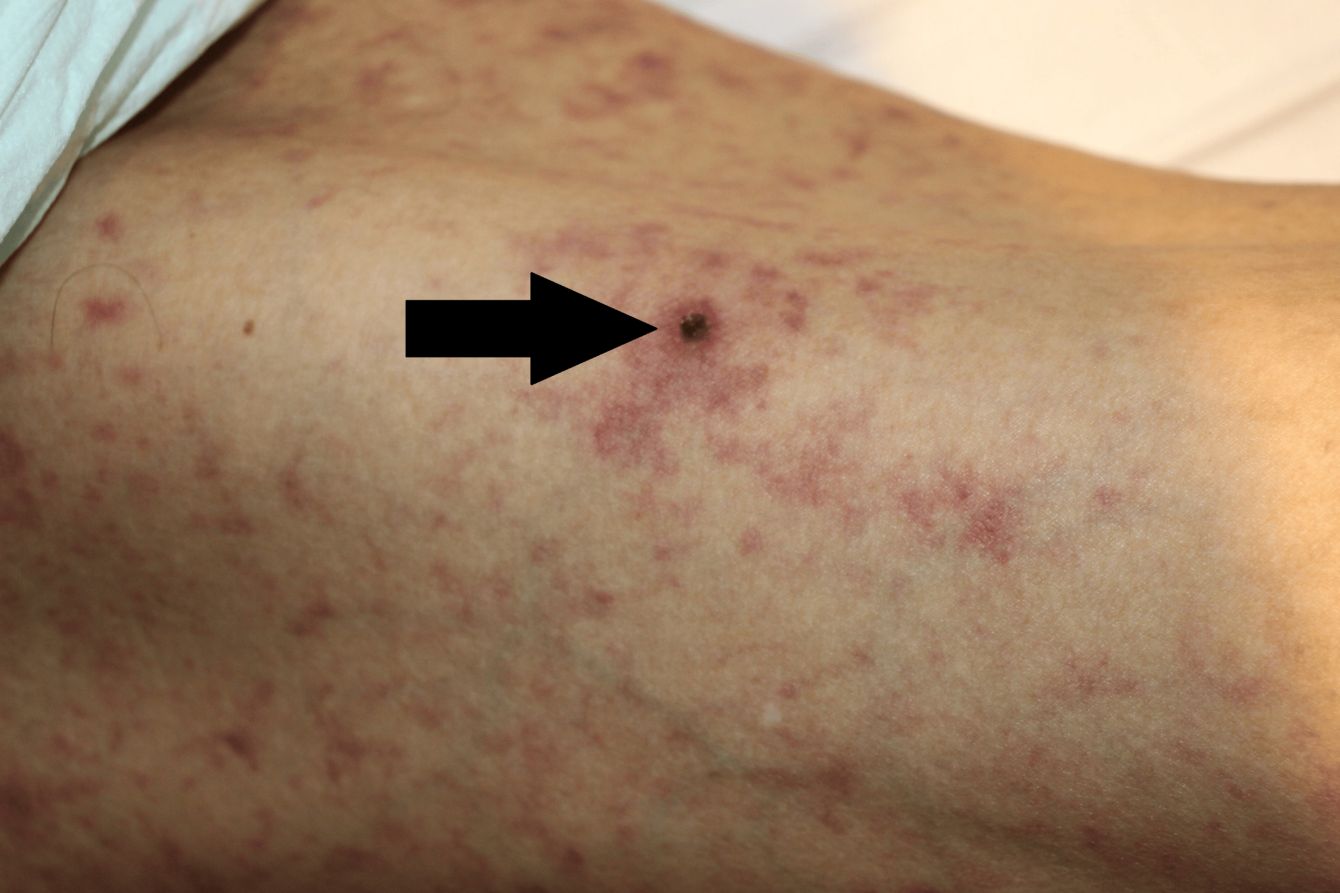
A scab suspected to be a tick bite on admission day A dark scab was observed on the right thigh, suspected to be an eschar resulting from a tick bite. Small eschar was typical for Japanese spotted fever.

The rash was initially attributed to COVID-19; however, after admission, a dermatological consultation was requested because its presence on the palms and soles was rare. A dermatologist noted that this finding was atypical for COVID-19 and suggested Japanese spotted fever. Additionally, an eschar, a scab suspected to result from a tick bite, was observed on the right thigh (Figure 4). A sample was sent for PCR testing, and minocycline was initiated.

Intravenous insulin with glucose was administered for hyperkalemia, successfully reducing serum potassium. Fluid therapy maintained the patient’s minimum necessary urine output, leading to a gradual decline in potassium levels. However, on hospital day 2, ventricular fibrillation (VF) occurred. Return of spontaneous circulation was achieved with basic life support alone. However, the patient subsequently developed hypotension, with systolic blood pressure in the 60s mmHg. Septic shock due to co-infection with COVID-19 and Japanese spotted fever was suspected. Although serum potassium had decreased to 5.8 mEq/L, VF was induced by mild hyperkalemia compounded by hypoperfusion. The patient was admitted to the intensive care unit and underwent continuous kidney replacement therapy (CKRT).

Continuous intravenous noradrenaline, vasopressin, and hydrocortisone were initiated for septic shock. CKRT rapidly reduced serum potassium levels, and urine output increased with circulatory stabilization, allowing discontinuation of CKRT on hospital day 3. Noradrenaline and hydrocortisone were continued until hospital day 6, while vasopressin was discontinued on hospital day 7. That same day, the patient developed fever and was started on meropenem for nosocomial pneumonia, with Serratia marcescens and Klebsiella pneumoniae detected in sputum cultures. PCR testing of the scab confirmed Japanese spotted fever, and minocycline was administered for one week. The patient was discharged from the intensive care unit on hospital day 8 and continued rehabilitation in the general ward. On hospital day 50, his activities of daily living were nearly fully independent. However, he was transferred to a convalescent rehabilitation hospital with the goal of discharging home.

## Discussion

Japanese spotted fever is a tick-borne infectious disease caused by Rickettsia japonica. The rash is typically small and primarily affects the extremities, including the palms and soles. The prognosis is favorable, with a mortality rate of 0.91-1.1%, compared to 5%-10% for Rocky Mountain spotted fever. Early administration of tetracyclines is crucial, and treatment should not be delayed while awaiting PCR test results [1, 2, 12].

COVID-19, caused by SARS-CoV-2, is characterized by fever and respiratory symptoms such as cough and shortness of breath. Renal failure is a common complication of COVID-19 and is associated with increased mortality [5]. A multicenter cohort study reported that 36.6% of hospitalized patients with COVID-19 developed acute kidney injury (AKI), and 14.3% required renal replacement therapy [13]. Although rash is not a primary symptom, it occurs in approximately 20% of patients, with various presentations, including urticaria, varicella-like vesicles, transient livedoid eruptions, livedoid vasculopathy, purpuric eruptions, lichenoid photodermatitis, erythroderma, photo-contact dermatitis, and generalized pustular figurate erythema. However, COVID-19-related rashes do not affect the palms and soles [11]. Although the rash of this case was not inconsistent with COVID-19 which was possible to cause various types of rash, the small erythema was more typical for Japanese spotted fever. Moreover, the rash on the palms and soles was atypical for COVID-19 and led to the diagnosis of Japanese spotted fever.

The global COVID-19 pandemic has altered healthcare providers' clinical approach. They now prioritize screening febrile patients for COVID-19 and often limit physical examinations to reduce patient contact time [4]. Consequently, some reports suggest that focusing on diagnosing COVID-19 in febrile patients may delay the identification of other diseases [6-8]. Additionally, co-infections with COVID-19 with other infectious diseases have been documented, and co-infection is a risk factor for worsening outcomes [5]. Severe cases have also been reported [14]. Several reports describe co-infections of COVID-19 with rickettsial diseases, including Rocky Mountain spotted fever [15] and scrub typhus [16]. In some cases, the presence of an eschar was pivotal for diagnosis. Distinguishing between different skin lesions is essential, as rickettsial infections typically present with an eschar, whereas COVID-19 can cause black, necrotic lesions due to vasculitis that may resemble an eschar [17, 18].

Cases of co-infection with COVID-19 and Japanese spotted fever have also been reported. In one instance, a patient developed hypotension during hospitalization, prompting further evaluation that led to the detection of an eschar and subsequent appropriate treatment [19].

In this case, rashes were initially ascribed to COVID-19. However, a dermatological examination enabled early diagnosis and treatment of Japanese spotted fever based on rash characteristics. The patient’s condition deteriorated rapidly, necessitating intensive care despite early treatment initiation. Delayed diagnosis could have resulted in a fatal outcome.

## Conclusions

This case illustrates how a diagnosis of Japanese spotted fever was initially overlooked due to a concurrent COVID-19 diagnosis. The rash on the palms and soles, along with eschar, were critical diagnostic clues. Despite initial deterioration, the patient recovered after antibiotic therapy and intensive care, and was transferred to a rehabilitation hospital. Once COVID-19 is diagnosed, healthcare providers may limit patient contact, making detailed examinations challenging. Even during a COVID-19 epidemic, thorough physical examinations remain essential for diagnosing coexisting infectious diseases.
